# Effects of the Yangjing Capsule Extract on Steroidogenesis and Apoptosis in Mouse Leydig Cells

**DOI:** 10.1155/2012/985457

**Published:** 2012-11-13

**Authors:** Dalin Sun, Yugui Cui, Baofang Jin, Xindong Zhang, Xiaoyu Yang, Chao Gao

**Affiliations:** ^1^Institute of Andrology, Nanjing University of Chinese Medicine, Nanjing 210046, China; ^2^State Key Laboratory of Reproductive Medicine, Clinical Center of Reproductive Medicine, and the First Affiliated Hospital, Nanjing Medical University, Nanjing 210029, China

## Abstract

*Objectives*. This study aimed to explore the effect and mechanism of Yangjing capsule on testosterone secretion in mouse Leydig tumor cells (MLTC-1). *Methods*. MLTC-1 cells were treated with the Yangjing capsule extract for 24 h. The testosterone level in medium was measured by radioimmunoassay. The expression of steroidogenic enzymes (StAR, CYP11A1, and HSD3B) in the cells was examined using real-time RT-PCR and immunoblotting. Additionally, MLTC-1 cells were treated for 48 h in a serum-free medium. The cell viability was measured by MTT assay. The cell cycle and apoptosis were analyzed using flow cytometry. The expression of activated caspase-3 was analyzed using RT-PCR and a colorimetric protease assay. *Results*. The Yangjing capsule extract increased testosterone production and the expression of StAR, CYP11A1, and HSD3B mRNAs and proteins compared with the control. H89 significantly inhibited these effects. The medicine improved the viability of MLTC-1 cells, decreased the number of cells in G0/G1 phase, and increased the number of cells in S-phase, as well as prevented cell apoptosis by inhibiting caspase-3. *Conclusion*. The Yangjing capsule can stimulate MLTC-1 cells to secrete testosterone and may be an alternative treatment for diseases characterized by insufficient testosterone production.

## 1. Introduction

Testosterone, the major circulating androgen, plays important roles in sexual differentiation, secondary sex characteristics, reproductive function, and sexual function [1]. Male hypogonadism, also known as testosterone deficiency syndrome (TDS), is often associated with impaired puberty, impotence, gynecomastia, and infertility or lowered spermatogenesis [2, 3]. Some men in midlife and old age experience late onset of hypogonadism (LOH). The incidence rates are 13% for men aged 40–49 years, 30% for men aged 50–59 years, and 47% for men over 70 years. Testosterone replacement therapy (TRT) may provide a wide range of benefits for hypogonadism, improving libido and sexual function, fertility, bone density, muscle mass, and quality of life [4, 5]. However, TRT involves the direct administration of an exogenous hormone. With this treatment, the androgen level in the serum is superphysiological and unstable. If injected with a testosterone oiling agent, the peak concentration of androgen in the serum is very high, and the hormonal fluctuation may be difficult for the patient to bear. Furthermore, TRT could increase the risk of prostate cancer, worsen the symptoms of benign prostatic hypertrophy, sleep apnea and congestive heart failure, cause liver toxicity, and promote gynecomastia, infertility, and skin diseases [4]. 

Formulations of traditional Chinese medicine, especially those intended for kidney support, have shown significant advantages for the treatment of male hypogonadism through multiple mechanisms of action as well as interactions with multiple targets, including opsonic action [6, 7]. The Yangjing capsule, which is composed of *Herba Epimedii Brevicornus, Radix Rehmanniae Preparata, Rhizoma Polygonati Sibirici, Placenta Hominis, Angelica sinensis*, and other components, primarily acts by stimulating the kidney. It has been suggested for the treatment of diseases of the reproductive system due to its endogenous effect on androgen production, including male infertility, sexual dysfunction, and LOH. In our previous study, we found that the Yangjing capsule improved sperm motility, concentration, and DNA integrity in infertile males, and it improved the libido and erections of patients with male sexual disorders [8–10]. We proposed that the Yangjing capsule may promote androgen synthesis and hormonal balance, which could be the basis for the treatment of male infertility and sexual dysfunction. 

Testosterone is primarily produced by testicular Leydig cells, which is regulated by LH [11]. By binding to the membrane LH receptor, LH induces cAMP synthesis in Leydig cells. cAMP catalyzes PKA synthesis, and PKA transports cholesterol from the cytoplasmic pool to the mitochondria to promote steroidogenesis by steroidogenic enzymes (CYP11A1, HSD3B, and so on) and StAR [12, 13]. Additionally, steroidogenesis is also dependent on the number and activity of Leydig cells [13]. The number of Leydig cells is maintained at equilibrium due to the balance between proliferation and apoptosis [14]. Excessive apoptosis may produce adverse effects, such as testosterone deficiency [13, 15, 16].

In this study, we explored the role of the Yangjing capsule in regulating steroidogenesis in Leydig cells and investigated its potential mechanism to provide evidence in supporting of treatment of male infertility and hypogonadism with the Yangjing capsule.

## 2. Materials and Methods

### 2.1. Chemicals

RPMI 1640 medium, fetal bovine serum and lyophilized trypsin-EDTA were obtained from GIBCO BRL (Grand Island, NY, USA). 3-[4,5-Dimethylthiazolyl-2]-2,5-diphenyltetrazolium bromide (MTT), dimethyl sulfoxide (DMSO), human chorionic gonadotropin (hCG), N-(2-[p-bromocinnamylamino] ethyl)-5-isoquinolinesulfonamide hydrochloride (H89), diethylpyrocarbonate (DEPC), sodium dodecyl sulfate (SDS), and Tris/HCl were purchased from Sigma (St. Louis, MO, USA). The caspase-3 colorimetric protease assay kit and the whole protein extraction kit were purchased from Keygen (Keygen Biotech. Co. Ltd., Nanjing, China). Trizol reagent, PrimeScript RT Master Mix, and SYBR Green PCR Master Mix reagent kits were obtained from TaKaRa (TaKaRa Biotechnology, Dalian, China). The primers were synthesized by Invitrogen Life Tech (Carlsbad, CA, USA). The goat polyclonal anti-CYP11A1 and rabbit polyclonal anti-HSD3B antibodies were purchased from Santa Cruz Biotechnology (Santa Cruz, CA, USA). The rabbit polyclonal anti-StAR antibody was obtained from Abcam (Cambridge Science Park, Cambridge, UK). The mouse polyclonal anti-GAPDH antibody and HRP-conjugated secondary antibodies were purchased from Bioworld (St.LouisPark,MN, USA). Enhanced chemiluminescence was obtained from Amersham Biosciences (Uppsala, Sweden).

### 2.2. The Yangjing Capsule Extract

The Yangjing capsule is composed of 11 traditional Chinese drugs: 13.3% *Herba Epimedii Brevicornus*, 6.7% *Rhizoma Polygonati Sibirici*, 6.7% Radix Rehmanniae preparata, 10% Radix Astragali Mongolici, 6.7% Placenta Hominis, 6.7% *Semen Astragali Complanati*, 10% *Radix Angelicae Sinensis*, 6.7% *Hirudo*, 6.7% *Semen Litchi*, 13.3% *Semen Vaccariae Segetalis*, and 13.3% *Concha Ostreae* (calcined). The Yangjing capsule extract was prepared based on the methods described by Kao et al. and Hu et al. [17, 18]. The content of the Yangjing capsule (3.33 g, equivalent to 10 g of crude drug) was extracted with 333 mL of distilled water and subsequently subjected to ultrasonic extraction for 45 min. The supernatant was collected. The precipitate was dissolved and extracted in a similar manner. The two solutions were combined and centrifuged at 13,000 g and 4°C for 30 min to collect the supernatant, which was concentrated to 100 mL with a rotary evaporator at 60°C. The final concentration of the Yangjing capsule extract corresponded to 100 mg/mL of the crude herbal dose. No hCG or estradiol was detected in the extract by radioimmunoassay (RIA), which could avoid the influence of hCG and estradiol contained in *Placenta Hominis* on the MLTC-1 cells. The pH of the extract was adjusted to 7.0, and the extract was sterilized by filtration on a super clean bench and stored at −70°C until use. 

### 2.3. Cell Culture and Treatment

The mouse Leydig tumor cells (MLTC-1) were derived from a transplantable Leydig cell tumor carried in C57BL/6 mice. MLTC-1 cells can produce testosterone with or without hCG. Therefore, MLTC-1 is a good cellular model for studying steroidogenesis and regulation. MLTC-1 cells were obtained from the Cell Institute of Shanghai (Shanghai, China) and cultured in RPMI-1640 medium that contained 2.05 mM L-glutamine, 10% heat-inactivated fetal bovine serum, 100 IU/mL penicillin, and 100 g/mL streptomycin in 5% CO_2_ at 37°C. The Yangjing capsule extract was diluted to low, medium, and high concentrations with RPMI-1640 without serum. The Yangjing capsule was administered to male infertility patients at a dose of 2 pills, tid. This dosage is equivalent to 9 g of crude drug. If the mean volume of one adult is approximately 0.06 m^3^, the distribution of medicine can be estimated as 0.15 mg/mL. Therefore, we chose the concentration of 0.1 mg/mL as the middle dose for treating the cells. Then, this dose was varied 10-fold to investigate a range of concentrations corresponding to 0.01, 0.1, and 1 mg/mL of the crude herbal dose. 

### 2.4. Hormone Assays

MLTC-1 cells were treated with the Yangjing capsule extract (0.01–1 mg/mL) over 24 h in 5% serum with or without hCG. The hCG group was used as a positive control. hCG can increase testosterone production through stimulation of the PKA signaling pathway in Leydig cells [19]. When we combined the Yangjing capsule extract with hCG, additive or synergistic effects were observed. To determine whether the Yangjing capsule extract acts through the PKA pathway to regulate steroidogenesis in MLTC-1 cells, the PKA pathway inhibitor H89 was used to treat cells for 24 h. The culture medium was subsequently collected, and testosterone was analyzed by radioimmunoassay (RIA). The minimum detectable concentration of testosterone was 0.2 ng/mL. The inter- and intra-assay coefficients of variation were <10% and <15%, respectively.

### 2.5. RNA Isolation and Quantitative Real-Time RT-PCR Analysis of Steroidogenic Enzymes

Cells at a density of 4 × 10^5^/well were plated in 6-well plates and treated with the Yangjing capsule extract in 5% serum for 24 h. Following stimulation by hCG for 4 h, the total RNA was extracted using Trizol reagent. The extracted RNA was measured by spectrometry at an OD_260/280_ and reverse transcribed into cDNA in a total volume of 20 *μ*L with PrimeScript RT Master Mix. All of the RT-PCR reactions were performed with an ABI Prism 7300 Sequence Detection System (Perkin Elmer Applied Biosystems, Foster City, CA, USA) using SYBR Green PCR Master Mix reagent kits. The housekeeping gene GAPDH was selected as the internal control. The primer sequences were as follows: GAPDH, sense: 5′-AGG TTG TCT CCT GCG ACT TCA-3′ and antisense: 5′-GGG TGG TCC AGG GTT TCT TAC T-3′, 186 bp; StAR, sense: 5′-CCA CCT GCA TGG TGC TTC A-3′ and antisense: 5′-TTG GCG AAC TCT ATC TGG GTC TG-3′, 142 bp; CYP11A1, sense: 5′-GAC CGA ATC GTC CTA AAC CA-3′ and antisense: 5′-GGA ACA TCT GGT AGA CAG CAT TG-3′, 278 bp; and HSD3B, sense: 5′-GTG GGG CTT CTG CCT TGA T-3′ and antisense: 5′-GGT TTT CTG CTT GGC TTC CTC-3′, 235 bp. The reactions were performed at 94°C for 3 min followed by 40 cycles at 94°C for 10 s, 60°C for 31 s, and 72°C for 30 s. A melting curve analysis was performed to confirm the products. The relative abundances of the target mRNAs were calculated using the 2^−ΔΔCt^ method. The data were expressed as the percentage of control (100%). 

### 2.6. Protein Extraction and Western Blot Analysis

Cells were seeded in 60 mm dishes at a density of 1 × 10^6^/well, and the Yangjing capsule extract in 5% serum was added for 24 h or for 4 h with activation of hCG. The cells were harvested, washed three times with precooled PBS, and treated with cell lysis buffer for Western blot analysis. After centrifugation at 12,000 g at 4°C for 20 min, the supernatants were collected and stored at −70°C until analysis. The concentrations of protein were determined using the bicinchoninic acid assay [20]. The proteins were normalized to 50 *μ*g/lane, separated by 12% sodium dodecyl sulfate polyacrylamide gel electrophoresis (SDS-PAGE), and subsequently transferred to nitrocellulose membranes. After treatment with blocking solution (5% skim milk powder in Tris-buffered saline) at 37°C for 1 h, the membranes were incubated overnight with the primary antibodies goat polyclonal anti-CYP11A1 (1: 500), rabbit polyclonal anti-StAR (1: 500), rabbit polyclonal anti-HSD3B (1: 500), or mouse polyclonal anti-GAPDH (1: 2000) at 4°C. After washing with TBS three times, the membranes were incubated with HRP-conjugated secondary antibodies (1: 5000) at 37°C for 1 h and examined using enhanced chemiluminescence. The relative protein levels in each sample were normalized to the levels of GAPDH to standardize the variations in loading. Densitometric analyses of the scanned immunoblotting images were performed using a Quantity One image system. The data are expressed as a percentage of the control (100%).

### 2.7. Cell Viability Assay

The viability and proliferation of the cells were assessed by the MTT assay, which is based on the reduction of MTT by the mitochondrial dehydrogenase of intact cells to form a purple formazan product [21]. The cells were seeded in a 96-well plate at a density of 5 × 10^3^/100 *μ*L and routinely incubated for 24 h at 37°C prior to use. After 24 h, the cells were treated with different concentrations (0.01–1 mg/mL) of the Yangjing capsule extract without serum for different time intervals (24, 48 and 72 h). After treatment, the media that contained the Yangjing capsule extract were carefully removed by aspiration. Subsequently, 20 *μ*L of 5 mg/mL MTT in cell culture medium was added to each well and incubated for 4 h. To dissolve the resulting formazan crystals, 150 *μ*L of DMSO solution was added to each well, and the plates were brachytely vibrated in an incubator for 10 min. The amount of formazan was determined by measuring the absorbance (*A*) value at a wavelength of 490 nm with respect to 630 nm using a microplate reader (Bio-Rad, CA, USA). 

### 2.8. Analysis of Cell Cycle and Apoptosis Using Flow Cytometry

For cell cycle analysis, 4 × 10^5^ cells/well were seeded in 6-well multidishes and incubated until they reached the logarithmic phase. The cells were rinsed twice with PBS and cultured with different concentrations (0.01–1 mg/mL) of the Yangjing capsule extract without serum for 48 h. They were subsequently trypsinized and centrifuged at 600 g at 4°C for 5 min. The supernatant was removed, and the cells were resuspended in 1 mL of PBS. An aliquot of 3 mL of cold ethanol was added, and the mixture was kept at −20°C for 24 h. After centrifugation at 600 g at 4°C for 5 min, the pellet was treated with 2 mg/mL RNase A at 37°C for 30 min and stained with 50 *μ*g/mL propidium iodide containing 0.1% Triton X-100 and 0.02 mg/mL EDTA. The percentage of cells in each stage of the cell cycle was determined using CellQuest software (Becton Dickinson, NJ, USA). The proliferation index (PI) was calculated using the following formula: *PI* = (*S* + *G*2/*M*)/(*G*0/*G*1 + *S* + *G*2/*M*).

 The procedure for cell apoptosis analysis was the same as above until the supernatant was removed and the cells were resuspended in PBS at a density of 1 × 10^6^/mL. The suspension was subsequently added to FITC-labeled Annexin V and stained with propidium iodide. Flow cytometric analysis was performed using a FACSAria flow cytometer (Becton Dickinson, NJ, USA).

### 2.9. Caspase-3 mRNA and Protein Activity Assay

Cells at a density of 4 × 10^5^/well were plated in 6-well plates and treated with the Yangjing capsule extract without serum for 48 h to activate caspase-3. Then, the RNA was extracted and reverse-transcribed. The conditions for the RT-PCR reactions were same as above. The primer sequences were as follows: GAPDH, sense: 5′-AGG TTG TCT CCT GCG ACT TCA-3′ and antisense: 5′-GGG TGG TCC AGG GTT TCT TAC T-3′, 186 bp; and caspase-3, sense: 5′-ATG GGA GCA AGT CAG TGG AC-3′ and antisense: 5′-CGT ACC AGA GCG AGA TGA CA-3′, 136 bp. The activity of the caspase-3 protein was detected using a caspase-3 colorimetric protease assay kit. The caspase-3 colorimetric assay was based on the hydrolysis of the peptide substrate acetyl-Asp-Glu-Val-Asp-p-nitroanilide (Ac-DEVD-pNA) by caspase-3, resulting in the release of the p-nitroaniline (pNA) moiety, which had a high absorbance at 405 nm [22]. The cells were seeded at a density of 1 × 10^6^/well in 60-mm dishes and treated with the Yangjing capsule extract without serum for 48 h to stimulate caspase-3 activity. The cells were harvested and washed three times with ice-cold PBS. After centrifugation at 1,000 g for 3 min, the cells were lysed by sonication on ice for 20 min in a chilled cell lysis buffer. Subsequently, the lysates were centrifuged at 10,000 g at 4°C for 1 min, and the supernatants were collected. The protein concentration was determined and adjusted for the cell lysis buffer. From each sample, 50 *μ*L of protein were solubilized in twofold reaction buffer. After incubation at 37°C for 4 h, the value for every sample was read at 405 nm in a microtiter plate reader.

### 2.10. Statistical Analysis

The data were expressed as the means ± SD from at least three independent experiments. Statistically significant differences between the groups were determined by one-way ANOVA followed by the least significance difference (LSD) or with *t*-test comparison procedure. The statistical significance was set at *P* < 0.05.

## 3. Results 

### 3.1. Effects of the Yangjing Capsule Extract on Testosterone Production with or without hCG

**Figure 1:**
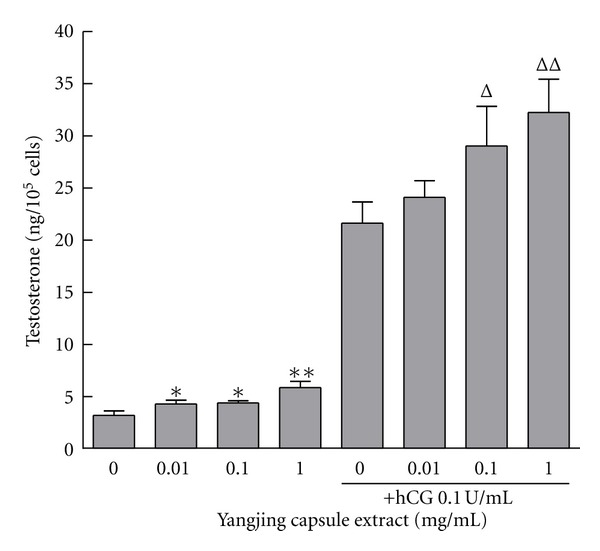
Effects of the Yangjing capsule extract on testosterone production in MLTC-1 cells. The Yangjing capsule extract (0, 0.01, 0.1, 1 mg/mL) was used to treat MLTC-1 cells for 24 h combined with or without hCG stimulation for 4 h. The results are given as the means ± SD from three independent experiments. Compared with the control without hCG, *P < 0.05, **P < 0.01; compared with the control treated only with hCG, ^Δ^ *P* < 0.05, ^ΔΔ^ *P* < 0.01.

MLTC-1 cells were treated with the Yangjing capsule extract for 24 h combined with or without 0.1 U/mL of hCG for 4 h (Figure 1). Testosterone production significantly increased after treatment with the Yangjing capsule extract at concentrations greater than 0.01 mg/mL without hCG (*P* < 0.05) and at concentrations greater than 0.1 mg/mL with hCG (*P* < 0.05) when compared with the controls.

### 3.2. Effects of the Yangjing Capsule Extract on the Expression of StAR, CYP11A1, and HSD3B mRNAs and Proteins

**Figure 2:**
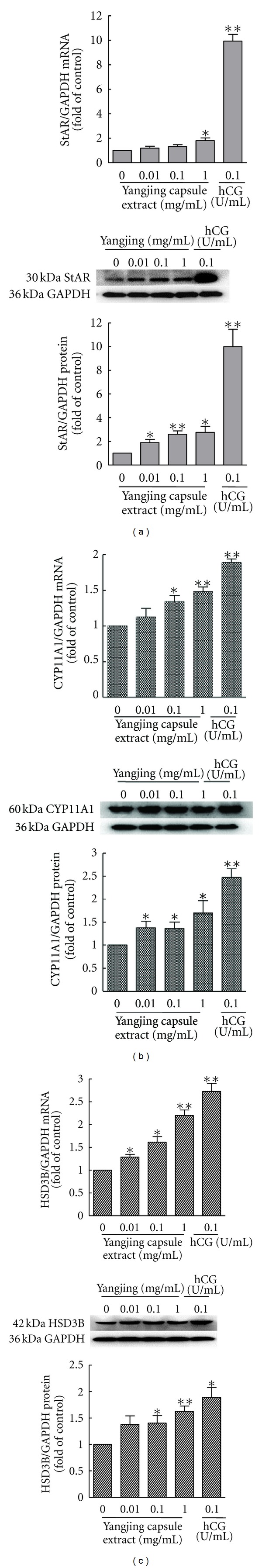
Effects of the Yangjing capsule extract on the expression of StAR (a), CYP11A1 (b), HSD3B (c) mRNAs and proteins in MLTC-1 cells. The Yangjing capsule extract was used to treat MLTC-1 cells for 24 h, and 0.1 U/mL hCG was added for 4 h. Expression of mRNAs was detected by real-time RT-PCR. Expression of proteins was detected by Western blot. The data are expressed as the percentage of the control (100%). The results are given as the means ± SD from three independent experiments. Compared with the control, *P < 0.05, **P < 0.01.

Three key enzymes (StAR, CYP11A1, and HSD3B) were chosen to examine the mechanism by which the Yangjing capsule extract regulates steroidogenesis.  Figure 2(a) showed that the expression levels of StAR mRNA and protein were increased significantly by the Yangjing capsule extract at concentrations of 1 mg/mL and greater than 0.01 mg/mL, respectively (*P* < 0.05).  Figure 2(b) showed that the expression of CYP11A1 mRNA and protein were markedly increased by the Yangjing capsule extract at doses of greater than 0.1 and 0.01 mg/mL, respectively (*P* < 0.05).  Figure 2(c) showed that the Yangjing capsule extract induced the expression of HSD3B mRNA and protein in a dose-dependent manner (*P* < 0.05). As expected, hCG stimulated the expression of StAR, CYP11A1, and HSD3B mRNAs and proteins. 

### 3.3. Effects of the Yangjing Capsule Extract on Testosterone Production Was Inhibited by H89

**Figure 3:**
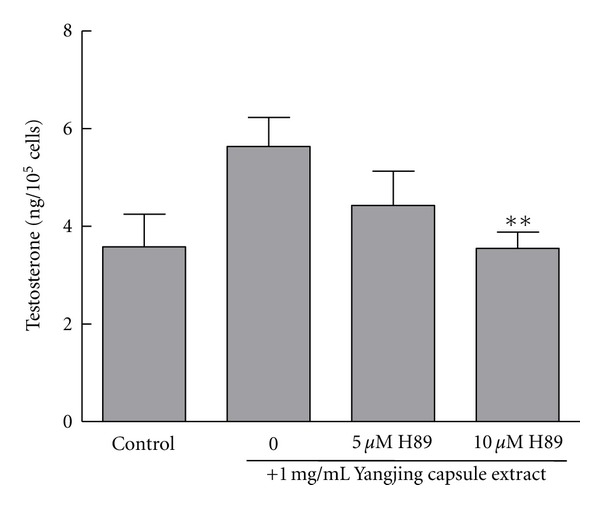
The effects of the Yangjing capsule extract on testosterone production were inhibited by PKA inhibitor H89. The Yangjing capsule extract was used to treat cells combined with or without 5 *μ*M and 10 *μ*M H89 for an incubation period of 24 h. The results are given as the means ± SD from three independent experiments. Compared with treatment with the Yangjing capsule extract alone, **P < 0.01.

To explore whether the Yangjing capsule extract acts on the PKA pathway, the H89, a PKA inhibitor, was added for an incubation period of 24 h. H89 (5 *μ*M and 10 *μ*M) caused a dose-dependent decrease in testosterone production that was stimulated by the Yangjing capsule extract (Figure 3). Moreover, H89 (10 *μ*M) significantly inhibited Yangjing-stimulated testosterone production by 37% (*P* < 0.01). 

### 3.4. Effects of the Yangjing Capsule Extract on Cell Viability

**Figure 4:**
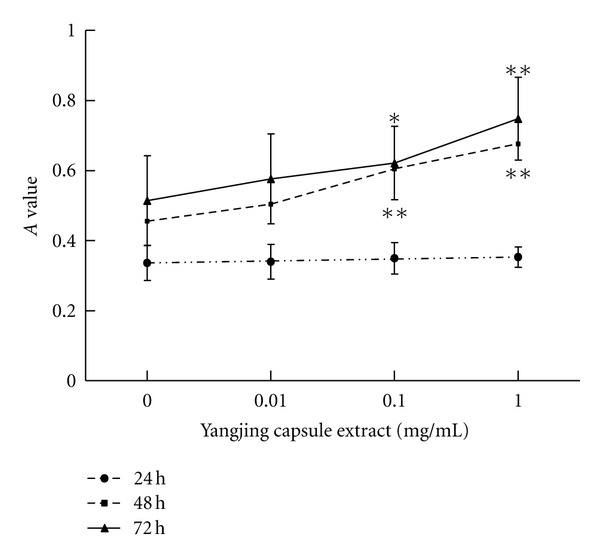
Effects of the Yangjing capsule extract on the viability of MLTC-1 cells. The Yangjing capsule extract was added to MLTC-1 for 24–72 h. Cell viability was analyzed by the MTT assay. The results are given as the means ± SD from three independent experiments. Compared with control, *P < 0.05, **P < 0.01.

The effect of the Yangjing capsule extract on the cell viability was both dose and time dependent (Figure 4). The absorption values significantly increased after treatment with the Yangjing capsule extract (0.1 and 1 mg/mL) for 48 h and 72 h (*P* < 0.05). The results showed that the Yangjing capsule extract improved the cell viability of MLTC-1 cells after treatment for 48 and 72 h at doses above 0.1 mg/mL. Therefore, we chose the time point of 48 h to observe the effect of the Yangjing capsule extract on the cell cycle and apoptosis.

### 3.5. Effects of the Yangjing Capsule Extract on Cell Cycle

**Figure 5:**
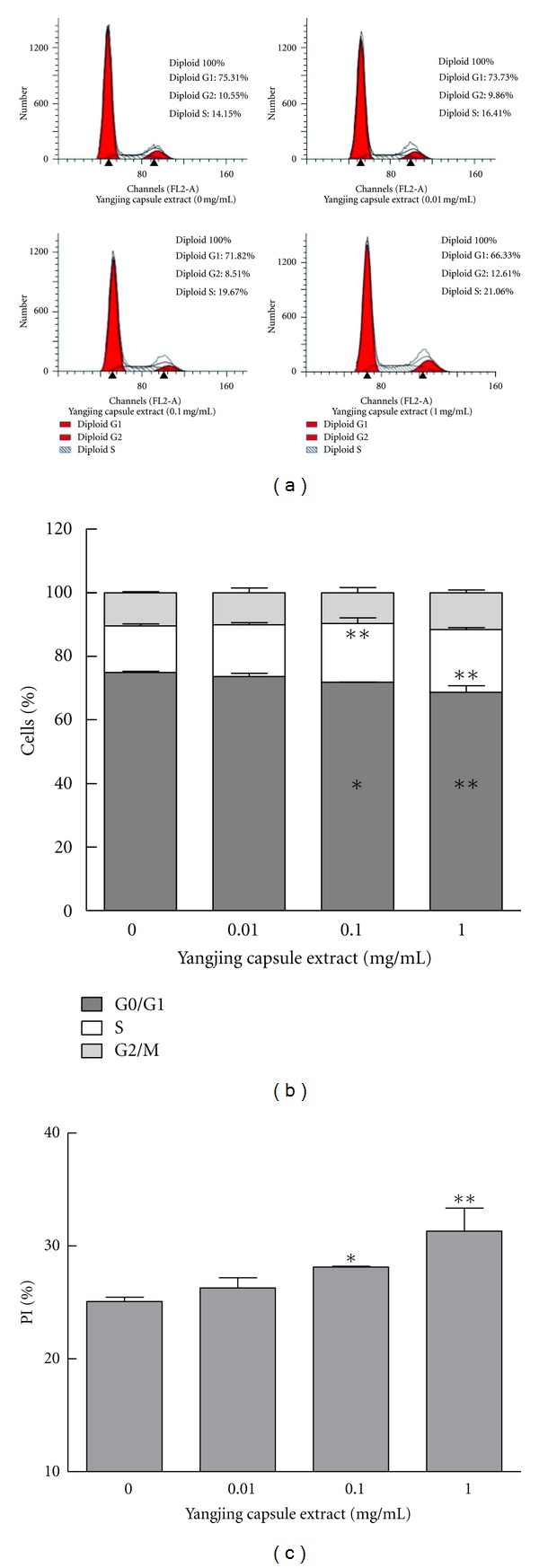
Effects of the Yangjing capsule extract on the MLTC-1 cell cycle. The Yangjing capsule extract was added to MLTC-1 cells for 48 h without serum. The cells were analyzed by flow cytometry. (a) The percentage of cells in each cell cycle stage was analyzed using flow cytometry after staining with propidium iodide. (b) The ratios of cells in the different phases are represented as percentage. (c) The effect of the Yangjing capsule extract on the PI of MLTC-1 cells. The results are given as the means ± SD from three independent experiments. Compared with control, *P < 0.05, **P < 0.01.

Flow cytometry analysis of MLTC-1 cells incubated with the Yangjing capsule extract for 48 h showed the changes in the percentage of cells at different stages, G0/G1, S, and G2/M (Figures 5(a) and 5(b)). The number of cells in G0/G1 phase significantly decreased, while the number of cells in S phase increased in groups treated with concentrations higher than 0.1 mg/mL (*P* < 0.05). However, the percentage of cells in G2/M phase showed no significant difference when compared with the controls (*P* > 0.05). In addition, the PI of MLTC-1 cells showed differences at concentrations higher than 0.1 mg/mL (Figure 5(c)). 

### 3.6. Effects of the Yangjing Capsule Extract on Cell Apoptosis and Caspase-3 Expression

**Figure 6:**
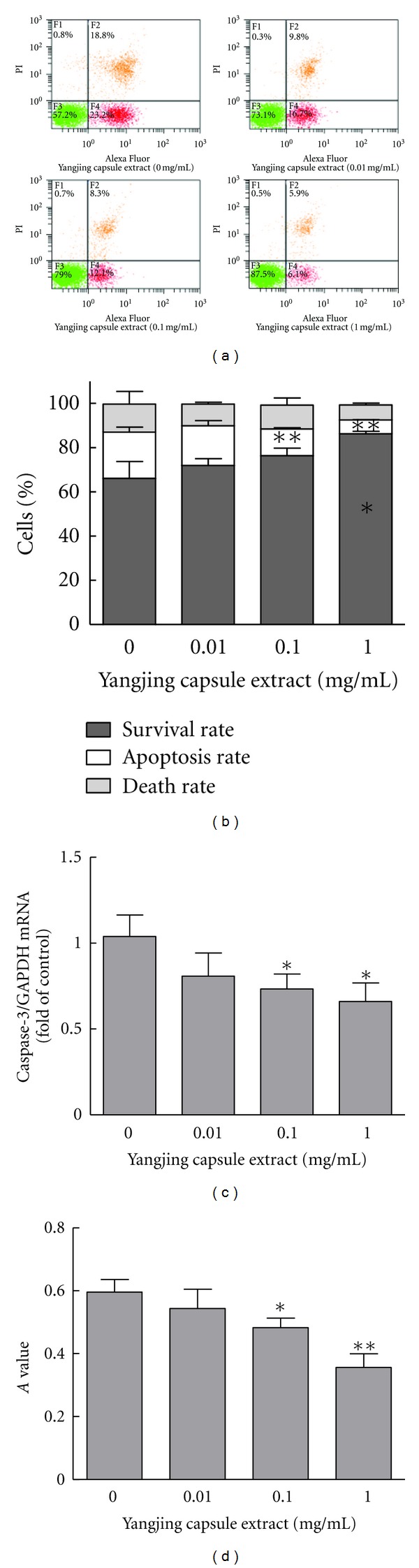
Effects of the Yangjing capsule extract on apoptosis and caspase-3 expression in MLTC-1 cells. The Yangjing capsule extract was added to MLTC-1 cells for 48 h without serum. The cells were collected and analyzed using flow cytometry. The expression of caspase-3 mRNA and protein was detected by real-time RT-PCR and a colorimetric assay. (a) The percentages of viable cells are shown in the lower left quadrant, and the percentages of apoptotic cells are shown in the lower right quadrant. (b) The ratios of the above cells are represented as percentages. (c) The effect of the Yangjing capsule extract on expression of caspase-3 mRNA. (d) The effect of the Yangjing capsule extract on caspase-3 protein activity. The determination of caspase-3 activity was performed using a caspase-3 colorimetric protease assay kit. The results are given as the means ± SD from three independent experiments. Compared with control, *P < 0.05, **P < 0.01.

To assess the effect of the Yangjing capsule extract on the apoptosis of MLTC-1 cells, the cells were treated for 48 h and examined using flow cytometry (Figure 6(a)). The apoptosis rate decreased after treatment with the Yangjing capsule extract, the survival rate increased (Figure 6(b)), and both changes were dose-dependent (*P* < 0.05 or *P* < 0.01). These results showed that the Yangjing capsule extract has a protective effect on MLTC-1 cells. 

The expression of caspase-3 is showed in Figures 6(c) and 6(d). The expression of caspase-3 mRNA and protein decreased after treatment with the Yangjing capsule extract for 48 h in a dose-dependent manner (*P* < 0.05). This result suggested that the Yangjing capsule extract inhibited apoptosis by suppressing caspase-3 expression.

## 4. Discussion

The Yangjing capsule has been used in the clinic for the treatment for male patients with infertility, LOH and sexual dysfunction. However, its mechanisms are still unclear. In this study, we found that the Yangjing capsule extract stimulated steroidogenesis and exerted a protective effect on Leydig cells. 

It has been widely accepted that many Chinese medicines intended for kidney support can also regulate steroidogenesis and hormonal balance [7, 23]. The Yangjing capsule extract increased testosterone production in MLTC-1 cells after 24 h of activation in a dose-dependent manner. Additionally, it affected testosterone production in concert with hCG. It is well known that LH and hCG are glycoproteins that contain polysaccharides [24]. Polysaccharides have been found in interstitial tissues as structural molecular forms that function by recognizing, connecting, or contacting molecules involved in cell interaction and communication [25]. Polysaccharides are also components of immunoglobulins, hormones, and enzymes [26]. The glycoprotein of LH/hCG plays an important role in association with the LH receptor to activate the signaling pathway for steroidogenesis [24]. It is possible that parts of the polysaccharides in the Yangjing capsule extract may be similar to LH in their structure and have the ability to recognize LH receptors on Leydig cells and then stimulate testosterone production. Moreover, it has been demonstrated that icariin, a flavonoid isolated from Herba Epimedii Brevicornus, has testosterone mimetic properties and therapeutic potential in the management of hypogonadism [27]. However, it remains to be elucidated whether these components participate in the critical function of steroidogenesis.

We also found that the Yangjing capsule extract significantly stimulated the expression of StAR, CYP11A1, and HSD3B mRNAs and proteins. hCG is well known to stimulate the cAMP-PKA signaling pathway to regulate steroidogenesis in Leydig cells *in vivo* and *in vitro*. We hypothesized that the Yangjing capsule induced testosterone production via the cAMP-PKA signaling pathway. This hypothesis was confirmed by the observation that H89, an inhibitor of the PKA pathway, significantly reduced the testosterone production induced by the Yangjing capsule extract. We can conclude that the Yangjing capsule extract most likely activated the cAMP-PKA signaling pathway to stimulate the expression of StAR, CYP11A1, and HSD3B, and then it increased testosterone production. 

In addition to steroidogenesis, the Yangjing capsule extract significantly decreased apoptosis in cultured MLTC-1 cells. Apoptosis in MLTC-1 cells was induced by serum-free medium. The Yangjing capsule extract could increase cell viability in a time- and dose-dependent manner. In the flow cytometry analysis, the Yangjing capsule extract significantly decreased the number of cells in G0/G1 phase, increased the number of cells in S phase, and significantly decreased the number of apoptotic cells. Meanwhile, it decreased caspase-3 expression in a dose-dependent manner. Therefore, the Yangjing capsule extract can exert a protective effect on Leydig cells *in vitro*. It is reported that polysaccharides exert antiapoptosis and antioxidative activities [28, 29]. It has been demonstrated that *Rhizoma Polygonati Sibirici* polysaccharides were able to prevent apoptosis in hypoxic neurons through upregulation of Bcl-2 protein expression, downregulation of Bax-2 protein expression and raising the ratio of Bcl-2/Bax [30]. *Radix Rehmanniae preparata* polysaccharides were clearly shown to promote the activity of SOD, CAT, and GSH-PX to perform anti-oxidant action [31], and they play a role in the nervous, endocrine, immune, and hematopoietic systems through their influence on cell proliferation and apoptosis [32].* Radix Astragali Mongolici* polysaccharides promote hematopoiesis and thrombopoiesis in a mouse model due to their anti-apoptosis activity [33]. Finally, *litchi* polysaccharides have the potential to enhance antioxidant activity [34]. These factors may be responsible for the anti-apoptosis action of the Yangjing capsule on MLTC-1 cells. Additional studies of the mechanism of the Yangjing capsule are necessary because this innovative traditional Chinese medicine has complex active ingredients.

Restoration of the endogenous hormonal balance and stimulation of testosterone production could be one of the mechanisms of Yangjing capsule when used to treat male hypogonadism, LOH, sexual dysfunction, and infertility. We found that the Yangjing capsule extract promoted testosterone production in Leydig cells through the activation of the cAMP-PKA signaling pathway, and it stimulated the expression of StAR, CYP11A1, and HSD3B. Endogenous testosterone exerts its action more effectively than exogenous androgen in treatment of LOH, avoiding the adverse side effects due to superphysiological and unstable hormone concentrations. In addition, we found that the Yangjing capsule extract had protective effects on Leydig cells. The increased apoptosis of Leydig cells is one of the pathophysiological causes of hypogonadism and LOH [13]. The testes of men with LOH exhibit testicular fibrosis, insufficient blood supply, and reduced quantities of Leydig cells [35]. The aged Leydig cells are mostly vacuolated, and multinucleated and contain abundant lipid droplets [36] due to the increased activation of apoptosis factors, including caspase-3 [37], and DNA fragmentation [38]. The Yangjing capsule decreased caspase-3 expression and decreased apoptosis in Leydig cells. Therefore, it represents a testicular protective treatment for LOH. 

In conclusion, we found that the Yangjing capsule extract effectively promoted testosterone production in Leydig cells while exerting protective effects on Leydig cells. The Yangjing capsule may be an alternative medicine for the treatment of diseases characterized by insufficient testosterone, such as male infertility, hypogonadism, and LOH. Further study will improve its clinical efficacy.
